# Educational expansion and inequalities in mortality—A fixed-effects analysis using longitudinal data from 18 European populations

**DOI:** 10.1371/journal.pone.0182526

**Published:** 2017-08-23

**Authors:** Olof Östergren, Olle Lundberg, Barbara Artnik, Matthias Bopp, Carme Borrell, Ramune Kalediene, Mall Leinsalu, Pekka Martikainen, Enrique Regidor, Maica Rodríguez-Sanz, Rianne de Gelder, Johan P. Mackenbach

**Affiliations:** 1 Centre for Health Equity Studies (CHESS), Stockholm University / Karolinska Institutet, Stockholm, Sweden; 2 Department of Public Health, Faculty of Medicine, University of Ljubljana, Ljubljana, Slovenia; 3 Epidemiology, Biostatistics and Prevention Institute, University of Zürich, Zürich, Switzerland; 4 Agència de Salut Pública de Barcelona, Barcelona, Spain; 5 Lithuanian University of Health Sciences, Kaunas, Lithuania; 6 Stockholm Centre for Health and Social Change, Södertörn University, Huddinge, Sweden; 7 Department of Epidemiology and Biostatistics, National Institute for Health Development, Tallinn, Estonia; 8 Department of Sociology, University of Helsinki, Helsinki, Finland; 9 Department of Preventive Medicine and Public Health, Universidad Complutense de Madrid, Madrid, Spain; 10 Department of Public Health, Erasmus MC, Erasmus University Medical Center, Rotterdam, The Netherlands; City University of New York (CUNY), UNITED STATES

## Abstract

**Objective:**

The aim of this paper is to empirically evaluate whether widening educational inequalities in mortality are related to the substantive shifts that have occurred in the educational distribution.

**Materials and methods:**

Data on education and mortality from 18 European populations across several decades were collected and harmonized as part of the Demetriq project. Using a fixed-effects approach to account for time trends and national variation in mortality, we formally test whether the magnitude of relative inequalities in mortality by education is associated with the gender and age-group specific proportion of high and low educated respectively.

**Results:**

The results suggest that in populations with larger proportions of high educated and smaller proportions of low educated, the excess mortality among intermediate and low educated is larger, all other things being equal.

**Conclusion:**

We conclude that the widening educational inequalities in mortality being observed in recent decades may in part be attributed to educational expansion.

## Introduction

Has the dramatic change in educational distribution in European populations contributed to widening social inequalities in health? A defining characteristic of Western societies during most of the post-war period has been substantive shifts in the educational distribution. Increasing proportions of the population now achieve tertiary education and diminishing proportions have only primary or lower secondary education, a process referred to as educational expansion [1, 2]. At the same time, relative inequalities in mortality between educational groups have widened [3]. It has been suggested that this widening is partly due to changes in the educational distribution, particularly the declining proportion of low educated is suggested to have increased negative selection into this group [4, 5]. Furthermore, familiar measures of health inequality, such as the relative index of inequality (RII) [6] or average intergroup difference (AID)[7], incorporate the educational distribution into the process of estimating health inequalities, implying that the two are connected. However, little empirical evidence exists on whether or not there is indeed a link between educational distribution and educational inequalities in mortality. This study attempts to empirically assess whether the educational distribution of the population may be related to the magnitude of educational inequalities in mortality.

**Fig 1:**
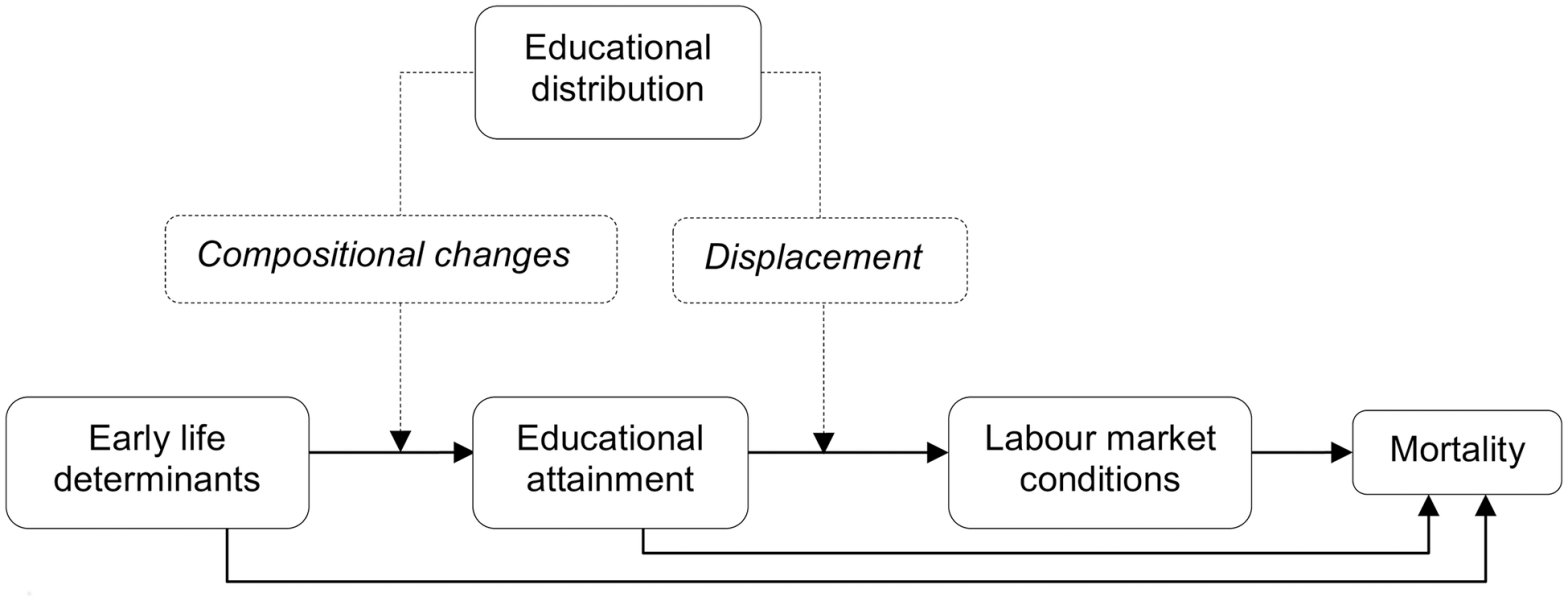
Conceptual model of suggested mechanisms.

We suggest two mechanisms that could generate an association between the educational distribution and the magnitude of educational inequalities in mortality, summarized in Fig 1. First, *compositional changes* may have occurred, i.e. mortality within educational groups may change as a result of changes in the composition of early life determinants common to individual educational attainment and adult health (Fig 1). A careful interpretation of the literature suggests that both individual characteristics and social origin are determinants of individual educational attainment [8–10]. Health in early life [5, 11] and other individual characteristics, for example cognitive ability [4], as well as childhood socioeconomic conditions [12, 13] are likely to influence both individual educational attainment and adult health independently. Analysing trends in intergenerational transmission of educational attainment in 42 nations in birth cohorts from the 1920s to the 1980s, Hertz et al. found that the absolute chances of high educational attainment increased across all social positions [2]. As successive cohorts have an increasing number of high educated these are increasingly recruited from a wider and thereby more heterogeneous range of backgrounds. Conversely, those who make up the low educated may increasingly consist of the most disadvantaged. It is then possible that the composition within each specific educational group, in terms of early life determinants common to individual educational attainment and adult health, changes during educational expansion in a way that dilutes the concentration of childhood advantage among the high educated and increases the likelihood of childhood disadvantage among the low educated. All other things being equal, this would lead to higher mortality in all educational groups, but given that the high educated have an advantage in terms of other resources available to them through education, this effect is most likely stronger among the low educated, leading to widening health gaps.

Second, educational expansion may lead to changes in the relative advantage and disadvantage on the labour market of having a higher and lower level of education, respectively. As the educational distribution shifts, the returns to educational investment in terms of labour market outcomes may change [14, 15]. If the increase in supply of highly educated labour is not matched by demand, the high educated may receive lower average returns in terms of income and occupational position as an increasing proportion of the population achieves high education. The low educated may also experience negative consequences since they often occupy the lowest positions on the labour market and may be pushed out of employment, a process referred to as *displacement* [16, 17]. To some degree, displacement may be temporary as the labour market may adjust to accommodate the supply of high skilled labour. Differences between educational groups in terms of working and labour market conditions account for an important part of the association between education and health [18–20]. While education, occupation, and income are correlated, they have been found to be independently associated with mortality [13–15] and educational inequalities in mortality are dependent on the distribution of income and occupational class within educational groups [16, 17]. Educational expansion may then change the employment rates, the matching of education and job qualifications as well as the average incomes within educational groups and thereby influence mortality within groups and differences in mortality across groups. Again, this mechanism, other things being equal, would lead to dilution of advantage among high educated and increased concentration of disadvantage among low educated. This would also lead us to expect increased inequalities in mortality.

The aim of this paper, then, is to empirically explore if the magnitude of educational inequalities in mortality is associated with the educational distribution as such.

We evaluate this by testing whether excess mortality by education is associated with the educational distribution. If the association between individual educational attainment and mortality is systematically different across educational distributions, this would suggest that educational expansion influences the magnitude of educational inequalities in mortality. We isolate the relationship between educational distribution and mortality using mortality data from a wide range of European populations across several decades, adjusting for variation in mortality by period and population. The proposed mechanisms outlined above suggest that both *compositional changes* and *displacement* contribute to a relative deterioration of health within all educational groups, but also that this tendency will most likely be stronger among lower educational groups. Therefore, we hypothesize that larger proportions of high educated and smaller proportions of low educated will be linked to wider relative educational inequalities in mortality.

## Materials and methods

**Fig 2:**
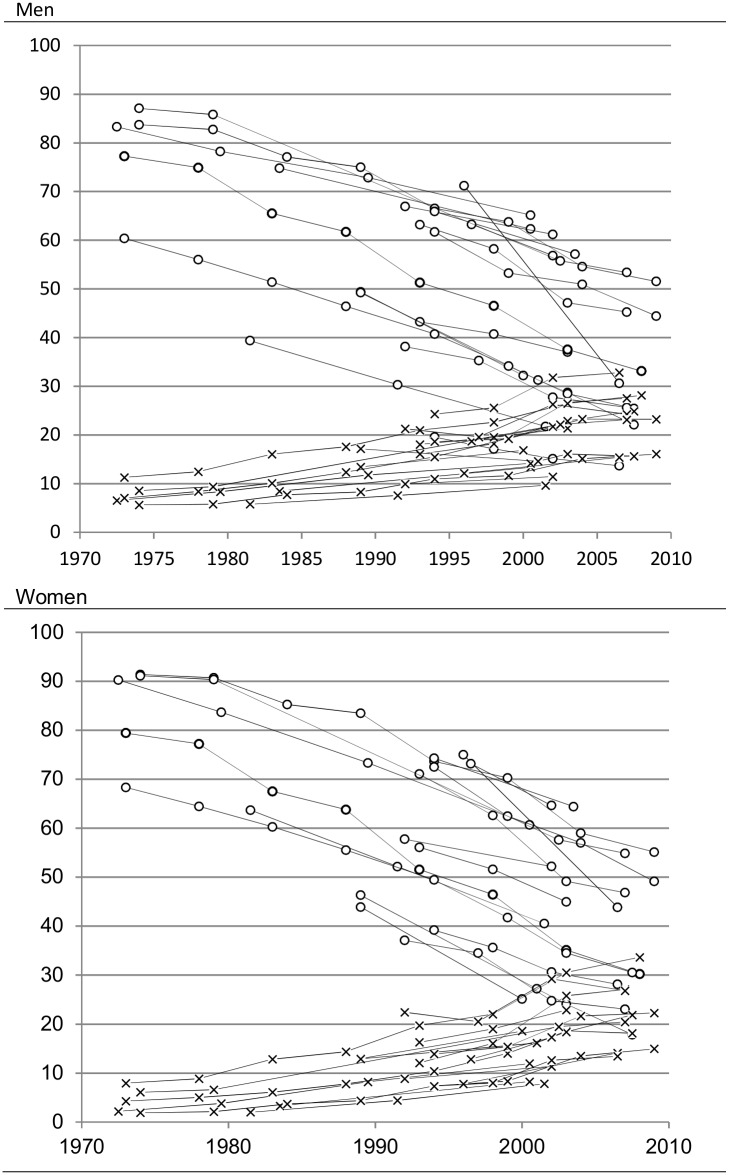
Observed average proportions of high and low educated by median year among men and women. Each data point represents one combination of population and period. Age standardized using the observed gender specific age distribution in the pooled data.

Data on 18 populations across 16 European countries were collected as part of the Demetriq project. Most data sets covered the entire national territories, except Italy for which data from the Turin region only was available, and Spain with data from the region of Madrid, the Basque Country and the city of Barcelona only. The length of time for which data were available varied between countries from 7 to 40 years. The data was divided into several periods within each population. Information on mortality was obtained from official mortality registries of each country. Most countries had longitudinal mortality data linked with population censuses except Czech Republic, Poland, Hungary and Estonia which had unlinked cross-sectional data (aggregated over a period of 5 years around a census year) and Barcelona (repeated cross-sectional data linked to the local census). In countries with linked data, educational information of the deceased was obtained by linkage of mortality registry data to the population census, which also provided educational information for the person-years at risk. In most countries linkage between the population and death registries was more than 95% complete; for countries where linkage failure exceeded 5% (Madrid, Barcelona and the Basque Country) mortality rates were multiplied by the inverse of the proportion of deaths that were successfully linked in order to adjust for the linkage failure. In unlinked data, educational information of the deceased was obtained from the mortality registry, while the educational level of the population at risk was obtained from the censuses. Educational level was recorded as the highest level of education completed. It was harmonized and reclassified into 3 categories based on the International Standard Classification of Education (ISCED-97): levels 0–2 (primary or lower secondary; “low”); levels 3–4 (upper secondary; “intermediate”) and levels 5–6 (tertiary; “high”). The proportion of the population aged 35–79 years with unknown educational attainment varied between 0 and 11%; subjects with missing information on education were excluded from the analyses. Due to inconsistencies in reporting education across periods or ages, some periods or ages were excluded, in total less than 0.5% of the total observations. Table 1 provides a specification of the populations, periods, and ages available. Fig 2 provides an overview of the changes in the average proportion of high and low educated for men and women within each population. As this study involved secondary analysis of routinely collected data, no ethics approval was necessary.

**Table 1:** Overview of available data, men and women 30–84 years.

Population	Period			
			Men	Women
Austria	1981–1982, 1991–1992,2001–2002	Person years	6 405 871	7 367 562
		Deaths	95 039	88 354
Belgium	1991–2001*, 2001–2009*	Person years	45 370 262	48 357 304
		Deaths	675 446	510 881
Czech Rep.	1982–1985, 1998–2003	Person years	27 695 126	31 219 382
		Deaths	519 117	436 655
Denmark^1^	1991–1995, 1996–2000,2001–2005	Person years	17 583 876	18 147 472
		Deaths	187 432	137 659
Estonia	1987–1991, 1998–2002	Person years	3 612 805	4 685 095
		Deaths	77 458	68 336
Finland	1970–1980*, 1980–1990*,1990–2000*, 2000–2010*	Person years	47 209 973	53 869 662
		Deaths	799 744	628 776
Hungary	1971–1974, 1978–1981,1988–1991, 1999–2002	Person years	44 169 144	51 450 844
		Deaths	986 760	810 991
Turin (Italy)	1971–1981*, 1981–1991*, 1991–2001*, 2001–2010*	Person years	9 274 527	10 926 773
		Deaths	141 257	112 440
Lithuania	1988–1990, 2000–2002,2001–2009*	Person years	12 040 297	15 215 345
		Deaths	248 268	181 128
Norway^2^	1970–1980*, 1980–1990*,1990–2001*, 2001–2009*	Person years	23 075 037	26 233 831
		Deaths	619 280	470 389
Poland^3^	1991–1993, 2001–2003	Person years	54 476 940	59 686 519
		Deaths	749 488	466 428
Slovenia^4^	1991–2001, 2002–2011	Person years	4 396 212	4 970 037
		Deaths	55 217	38 449
Barcelona (Spain)	1992–1996, 1997–2001,2002–2006, 2007–2010	Person years	9 045 886	10 622 124
		Deaths	123 954	87 978
The Basque C. (Spain)	1996–2001, 2001–2006	Person years	6 571 456	7 044 923
		Deaths	73 748	43 724
Madrid (Spain)	1996–1997, 2001–2003	Person years	4 566 588	5 229 947
		Deaths	54 436	35 293
Sweden^5^	1990–1999*, 2000–2008*	Person years	40 261 612	41 099 108
		Deaths	407 411	276 500
Switzerland	1990–2000*, 2000–2008*	Person years	25 001 645	28 826 491
		Deaths	32 5192	246 285
England and Wales	1971–1981*, 2001–2009*	Person years	2 242 214	2 526 616
		Deaths	37 647	31 213

* Period split into two during analysis

^1^ Available ages: 1991–1995: ages 30–69, 1996–2000: ages 30–74, 2001–2005: ages 30–84

^2^ Available ages: 50–84

^3^ Available ages: 1991–1993: ages 30–64, 2001–2003: ages 30–84

^4^ Available ages: 35–79

^5^ Available ages: 1990–1994, 2000–2004: 30–79, 1994–1999, 2004–2009: 30–84

If the educational distribution itself is associated with the magnitude of educational inequalities in mortality, we expect the relationship between individual educational attainment and mortality to be different across different educational distributions. There are several things to consider when estimating this relationship. First, both educational distribution and the magnitude of educational inequalities in mortality vary across age, making it difficult to assess whether the two are associated in a single population and period. By combining data from several populations and time points, we introduce variation between age and educational distribution, allowing adjustment for age and educational distribution simultaneously. However, mortality varies across populations and time, and using mortality data from several populations and periods introduces variation in absolute levels of mortality. Due to the fact that educational expansion and declines in mortality take place simultaneously, the absolute level of mortality is correlated with educational distribution; furthermore, lower absolute mortality risks tend to be associated with higher relative differences between groups. In order to isolate the relationship between educational distribution and inequalities in mortality, adjustment for the absolute level of mortality in different populations and across period is then necessary.

In order to adjust for between-population variance and time trends in mortality, we adopted a fixed-effects approach using each combination of population and period as a fixed effect. Fixed-effects models have been suggested as an alternative to multilevel models in cross-national analysis [21], as multilevel models can produce biased estimates when the number of higher-order observations are low [22]. Similar to multi-level models, the fixed-effects approach controls for higher-order variance, but does not allow inference on specific higher-order covariates [21]. Allison and Waterman [23] demonstrated that the unconditional negative binomial model, using a set of dummies to estimate fixed-effects and robust standard errors, is an unbiased fixed-effects estimator [23]. Consequently, we applied negative binomial regression using number of deaths as the outcome and person years at risk as the offset. Proportions of high and low educated were assessed for each five year age category and for men and women and separately. This was done for both technical and substantive reasons. The age-specific educational distribution varies within the fixed-effects and using age- and gender-specific measures improves statistical power by capturing the full variation in educational distribution. Measuring the age and gender-specific educational distributions also more accurately reflects the situation on the labour market where individuals are more likely to compare and compete with other individuals of similar age, and of the same gender as men and women, to some degree, tend to hold different occupations.

The final models included dummy variables for individual education (omitting high educational attainment, as that was used as the reference category), age, the proportion of high and low educated, interaction terms between individual intermediate education and educational proportions and between individual low education and educational proportion and a set of dummy variables for each combination of population-period. In all models, number of deaths was the outcome and person-time at risk was the offset. All models were stratified by gender. The final model may be described;

$$\begin{array}{l} ln\left(\mu_{ijk}\right)=\alpha \;+\;\beta_{i}POP\_PERIOD_{i}\;+\;\gamma_{j}EDU_{j}\;+\;\delta_{k}AGE_{k}\;+\;\theta LOW\_PROP_{ik}\;+\;\pi_{j}LOW\_PROP_{ik}\\ \;\times \;EDU_{j}\;+\;\vartheta HIGH\_PROP_{ik}\;+\;\rho_{j}HIGH\_PROP_{ik}\;\times \;EDU_{j}\;+\;ln\left(t_{ijk}\right)\;+\;\varepsilon_{ijk} \end{array}$$

where *i* = 1,…,67 (population-period), *j* = 1,2 (education) and *k* = 1,…,10 (age) and *POP_PERIOD* is the combination of population and period, *EDU* is educational attainment, *AGE* is the five-year age-group, *LOW_PROP* is the proportion of low educated, *HIGH_PROP* is the proportion of high educated, and *t* is person years of exposure time.

By including interaction terms between individual educational attainment and educational distribution, the model detects whether the association between individual educational attainment and mortality is systematically different across different educational distributions. Since the high educated are set as the reference category, the interaction terms may be interpreted as the association between excess mortality among the low and intermediate educated and educational distribution.

In order to assess how the educational distribution is related to educational inequalities in mortality we calculated expected mortality rate ratios and temporary life expectancies (the expected number of years lived within a set interval [24]) between 30 and 84 in three educational groups using estimates obtained in the regression models. Mortality rate ratios and temporary life expectancy were estimated in three scenarios based on the gender specific mean proportions and standard deviations observed when pooling all included data. The scenarios are defined in order to reflect the process of educational expansion. Scenario 1 was defined as the proportion of low educated being one standard deviation above the mean and the proportion of high educated one standard deviation below the mean. Scenario 2 was defined as the mean proportions of high and low educated. Finally, Scenario 3 was defined as the proportion of low educated being one standard deviation below the mean and the proportion of high educated one standard deviation above the mean (exact values may be found in Table 2). This method takes into account the different distribution of education and rate of change in distribution among men and women.

**Table 2:** Observed average proportion and standard deviation of low and high educated in pooled data, definition of scenarios used to estimate educational inequalities mortality in different educational distributions, men and women.

	Men		Women	
	Education			
	Low (%)	High (%)	Low (%)	High (%)
Avg.	55.2	15.7	59.3	12.4
SD	9.2	3.5	15.7	6.1
Scenario 1	64.4	12.3	75.0	6.3
Scenario 2	55.2	15.7	59.3	12.4
Scenario 3	46.2	19.2	46.7	18.5

## Results and discussion

Table 3 shows the results from the fixed-effects model. The coefficients express the average difference in ln death risk by one observed standard deviation in the proportion of high and low educated respectively. The upper part of the table presents the coefficients for individuals with high (reference), intermediate and low education respectively, showing that mortality is higher among the mid and low educated than among the high educated in both analyses, among men and women alike. The coefficients for “%low” and “%high” show the association between the educational distribution and mortality among the reference category, i.e., the high educated. The table also presents four interaction terms; the interaction between “%low” and “%high”, respectively, and individual level intermediate or low education. The interaction terms may be interpreted as the association between excess mortality by education and the proportion of individuals with intermediate and low education respectively.

**Table 3:** The association between individual education, proportion of high and low educated and all-cause mortality for men and women, 30–84 yrs.

		Men		Women	
		Coef.	p.	Coef.	p.
Education	High	0	ref.	0	ref.
	Intermediate	0.812	<0.001	0.236	<0.001
	Low	0.928	<0.001	0.526	<0.001
High education	% high	-0.094	<0.001	-0.040	0.002
	% high ×Int.	-0.018	0.068	0.023	0.043
	% high ×Low.	0.013	0.180	0.073	<0.001
Low education	% low	-0.093	<0.001	-0.151	<0.001
	% low ×Int.	-0.075	<0.001	-0.036	0.003
	% low ×Low	-0.073	<0.001	-0.071	<0.001

Negative binomial regression, controlled for age and population-period fixed effects. Coefficients for the proportion of high and low educated are given for one observed standard deviation difference in the gender specific average of proportion of low educated in pooled data.

Among men, the proportion of high educated was negatively associated with mortality among high educated, indicating that mortality among the high educated was lower when the proportion of high educated was larger. As indicated by the interaction terms, however, there was no association between the proportion of high educated and excess mortality by education. The results indicated that while the mortality tended to be lower among men at larger proportions of high educated, there is no association between the proportion of high educated and the level of excess mortality by education among men. This finding does not necessarily imply that there was no association between educational distribution and excess mortality by education. The proportion of low educated tend to decline as the proportion of high educated increase, both proportions need to be considered.

The proportion of low educated was negatively associated with mortality among high educated men, and the interaction terms indicated that excess mortality by education was negatively associated with the proportion of low educated. These results indicate that mortality tends to be lower in all educational groups at higher proportions of low educated, and that a smaller proportion of low educated is associated with larger excess mortality by education among men.

Among women, the proportion of high educated is negatively associated with mortality among the high educated, but positively associated with excess mortality among the intermediate and low educated. The results indicate that a larger proportion of high educated is associated with larger excess mortality by education among women.

The proportion of low educated is negatively associated with mortality among the high educated and negatively associated with excess mortality. A smaller proportion of low educated is associated with larger excess mortality by education among women.

**Fig 3:**
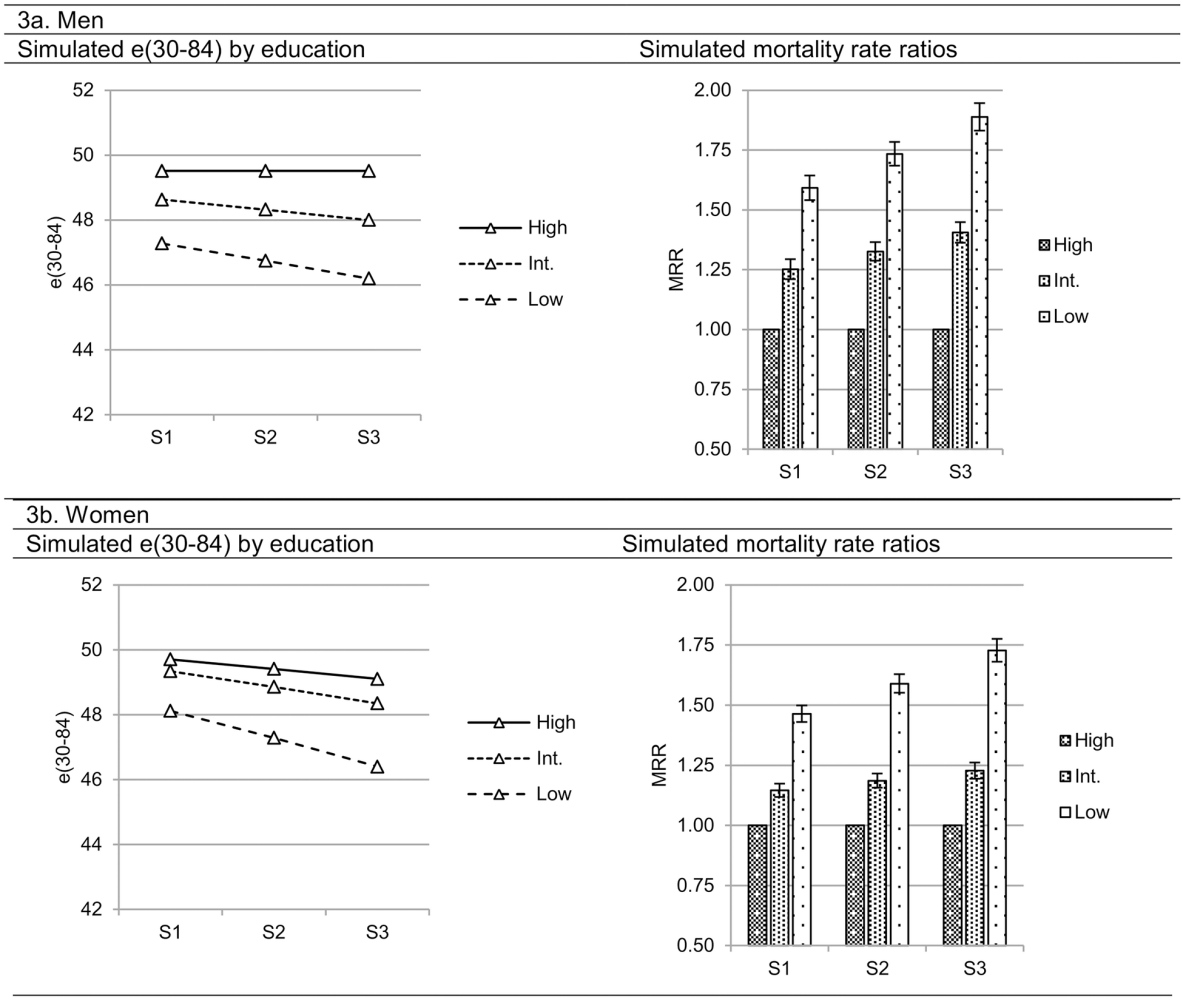
Simulated life expectancies between 30 and 84 years and mortality rate ratios at Scenario 1, 2, and 3, men and women.

The results indicate that educational distribution is associated with the magnitude of relative educational inequalities in mortality. A smaller proportion of low educated was associated with higher excess mortality among both men and women and a larger proportion of high educated was associated with higher excess mortality among women, but not among men. However, as Fig 2 demonstrates, the increase of the proportion of high educated and the decrease of the proportion of low educated tend to take place simultaneously, and the association between educational distribution and educational inequalities in mortality may be better illustrated when both proportions are taken into account simultaneously. Therefore, we estimated temporary life expectancies and mortality rate ratios based on the regression coefficients presented in Table 3 in three scenarios reflecting the progress of educational distribution. Fig 3 presents temporary life expectancies by education and mortality rate ratios simulated in three scenarios based on the observed mean and standard deviation of the proportion of high and low educated obtained from pooled data (see Table 2). Scenario 1 to 3 represents the process of educational expansion, with progressively smaller proportions of low educated and progressively larger proportions of high educated, all other things being equal.

Among men, simulated life expectancies among the intermediate and low educated tended to be lower in Scenario 2 and 3 compared to Scenario 1. Life expectancy among the high educated were similar across the three scenarios. The mortality rate ratios (MRR) tended to be larger in Scenario 2 and 3.

Among women, simulated life expectancies were lower in all educational groups in Scenario 2 and 3 compared to Scenario 1. The life expectancy among the high educated varied the least and the life expectancy in the low educated varied the most. Consequently, the pattern in MRR was similar among women, compared to men, with larger inequalities in Scenario 2 and 3 compared to Scenario 1.

Overall, the results among men and women are similar with the largest inequalities being observed in Scenario 3 and the smallest in Scenario 1.

### Summary of findings

The empirical analysis indicated that educational distribution *per se* is associated with the magnitude of educational inequalities in mortality. The results suggest that in populations with larger proportions of high educated and smaller proportions of low educated, the excess mortality among intermediate and low educated is larger, all other things being equal. Our findings clearly indicate that health inequalities are larger at lower proportions of low educated and higher proportions of high educated, both among men and women.

### Strengths and limitations

It should be noted that we here have made an attempt to study the importance of educational expansion *net of* differences in mortality between age-groups, period and countries. As the results are de-contextualised, they should be interpreted carefully in terms of policy and the specific national and temporal conditions need to be taken into account in order to formulate efficient strategies to narrow health inequalities. Though the simulated temporary life expectancies tended to be lower at larger proportions of high educated and lower proportions of low educated, this is not an indication that educational expansion is associated with shorter life expectancies In fact, during the period life expectancy increased in all educational groups in most societies [25, 26]. The simulated numbers are net of time trends, and during the period studied, life expectancies increased in most populations, and in most educational groups.

We performed a series of sensitivity analyses using regional subsets of populations (S1 Table) and random effects models allowing the association between education and mortality to vary by population and age. All models yielded similar patterns in terms of significance and direction especially among women, while point estimates differed across alternative model specifications. The coefficient point estimates should be interpreted carefully. Due to the smaller data sets, the proportion of high and low educated was tested in separate models in the regional subsets.

### Interpretation

From the literature, we identified two mechanisms potentially linking educational inequalities in mortality to educational expansion: *compositional changes* in early life determinants common to individual educational attainment and adult mortality and *displacement*; changes in the labour market outcomes of specific levels of education. We hypothesized that both mechanisms might result in a relative deterioration of health within all educational groups, but also that this tendency would most likely be stronger among lower educational groups, and hence that both mechanisms would be linked to increasing relative inequalities.

The two mechanisms proposed, i.e. compositional changes and displacement, operate under a set of assumptions. *Compositional changes* may influence the level of mortality within educational groups if there are any early life determinants common to educational attainment and adult health, and if educational achievement in absolute terms has increased across the entire population. *Displacement* may influence mortality within educational groups if educational distribution is associated with changes in labour market conditions in terms of occupation, income, and employment rates within educational groups, and if these factors are independently associated with mortality. Whether or not these assumptions are met is in principle an empirical question. While the literature seems to support the assumptions on a general level, national variations in the associations and their time trends are most likely to exist. Furthermore, the two mechanisms are likely taking place simultaneously and may be related. For example, having an advantageous social background or high cognitive ability may influence the returns to educational investment in terms of labour market conditions. The results from this study apply to tendencies on a European level, which should be considered when interpreting the findings of this study in national contexts.

In order to explore the possible role of displacement we performed an additional analysis adjusting the final model for occupational class, measured using five categories (non-manual, manual, farmers, self-employed, and missing or non-active). After adjusting for occupational class, the interaction coefficients were smaller but remained unchanged in terms of direction and statistical significance (S2 Table). These additional results indicate that labour market outcomes partly account for the association between educational distribution and excess mortality by education. The results could then be interpreted as providing some support for displacement contributing to the observed patterns. Due to the somewhat crude measure of occupational class and lack of information on income, we are likely not able to capture the full variation of labour market outcomes. Furthermore, as information on childhood conditions was not available for analysis, we could not directly test the role of compositional changes. These results should then be interpreted carefully and further research using more detailed data is needed in order disentangle the different processes.

The results among men shows that mortality tended to be higher within the intermediate and low educated at lower proportions of low educated and higher proportions of high educated whereas mortality among the high educated were stable across different educational distributions. Educational expansion has been taking place at a slower pace among men compared to women, and it is possible that because of this, the labour market outcomes and conditions among men have remained more stable compared to women. The process of displacement, where the high educated compete with other educational groups for positions on the labour market, may then be less pronounced among men. This is consistent with the finding that the proportion of high educated is not associated with excess mortality by education among men. The proportion of low educated was associated with excess mortality, which could be interpreted to indicate that the concentration of disadvantage in terms of common determinants of education and mortality increased among the low and intermediate educated. It is possible that education is a protective factor and resources made available through education may limit the health impact of increasing heterogeneity in terms of early life determinants and deteriorating labour market conditions [27, 28] among high educated men.

Among women, both lower proportions of low educated and higher proportion of high educated are associated with higher mortality within all educational groups. The results are then compatible with both compositional changes, and with displacement. Both men and women may experience health benefits of education as such. Education could to some degree compensate for the dilution of advantage and worse labour market conditions among the high educated through for example ability to navigate the medical system, social capital, and resource allocation [27, 28]. Since educational expansion has progressed faster among women, displacement could be more pronounced and it is possible that the benefits of education may not compensate for increasing heterogeneity and comparatively worsening labour market conditions to the same extent among women as among men. Furthermore, high educated women may increasingly seek employment in traditionally male dominated-sectors which could indicate that high educated women face additional obstacles on the labour market. Women may then receive lower average returns to educational investment than men, and may occupy jobs with lower status or less pay, as the competition becomes harder. Educational expansion has taken place simultaneously with rising levels of female labour market participation rates [29] and educational upgrading of traditionally female occupations [30], making it less straightforward to compare the relation between educational expansion and labour market outcomes between men and women.

Educational expansion could also indicate that low educated individuals increasingly fall short in social comparisons, which may induce further stress among the low educated. Having a level of educational attainment lower than what is deemed socially acceptable in a society, sometimes referred to as educational poverty [31], could lead to stigmatization [17]. As average educational attainment increases the low educated may be increasingly disadvantaged not only because of the composition of the group but also in terms of how this group is perceived and thereby treated.

## Conclusions

The results suggest that educational expansion, taking place over the last decades, may in itself be linked to the observed increase in relative educational inequalities in mortality. To what extent this is driven by processes based on childhood factors or factors linked to the labour market value of different educations need further analysis.

## Supporting information

S1 Table The association between individual education and proportion of low and high educated on all-cause mortality for men and women in regional subsets, 30–84 yrs. Negative binomial regression, controlled for age and population-period fixed effects. Coefficients for the proportion of low educated are given for a 10% difference in the proportion of low and high educated.
(DOCX)

S2 Table The association between individual education and proportion of high and low educated on all-cause mortality for men and women, 30–84 yrs. Adjusted for occupational class.
(DOCX)
