# Supplementary material for: Educational expansion and inequalities in mortality—A fixed-effects analysis using longitudinal data from 18 European populations
Source: PLoS One. 2017 Aug 23;12(8):e0182526. doi: 10.1371/journal.pone.0182526 (PMC5568384; doi:10.1371/journal.pone.0182526)
Supplement: S1 Table — Negative binomial regression, controlled for age and population-period fixed effects. Coefficients for the proportion of low educated are given for a 10% difference in the proportion of low and high educated. (DOCX) [file pone.0182526.s001.docx]

**S1 Table. The association between individual education and proportion of low and high educated on all-cause mortality for men and women in regional subsets, 30-84 yrs.**

|  |  |  | % low educated |  |  |  | % high educated |  |  |  |
| --- | --- | --- | --- | --- | --- | --- | --- | --- | --- | --- |
|  |  |  | Men |  | Women |  | Men |  | Women |  |
| Populations |  |  | Coef. | p. | Coef. | p. | Coef. | p. | Coef. | p. |
| Denmark, Finland, Norway, Sweden | Education | High | 0 | ref. | 0 | ref. | 0 | ref. | 0 | ref. |
|  |  | Int. | 0.708 | <0.001 | 0.346 | <0.001 | -0.123 | 0.040 | 0.036 | 0.312 |
|  |  | Low | 0.991 | <0.001 | 0.877 | <0.001 | -0.019 | 0.748 | 0.168 | <0.001 |
|  |  |  |  |  |  |  |  |  |  |  |
|  |  | % | 0.075 | <0.001 | -0.063 | <0.001 | -0.291 | <0.001 | 0.127 | <0.001 |
|  |  |  |  |  |  |  |  |  |  |  |
|  | Interaction | %*Int. | -0.084 | <0.001 | -0.035 | <0.001 | 0.217 | <0.001 | 0.069 | <0.001 |
|  |  | %*Low | -0.093 | <0.001 | -0.076 | <0.001 | 0.288 | <0.001 | 0.171 | <0.001 |
| Czech Rep., Estonia, Hungary, Lithuania, Poland | Education | High | 0 | ref. | 0 | ref. | 0 | ref. | 0 | ref. |
|  |  | Int. | 0.740 | <0.001 | 0.524 | <0.001 | -0,028 | 0.802 | 0.045 | 0.464 |
|  |  | Low | 1.333 | <0.001 | 1.253 | <0.001 | 0.044 | 0.700 | 0.054 | 0.380 |
|  |  |  |  |  |  |  |  |  |  |  |
|  |  | % | -0.152 | <0.001 | -0.099 | <0.001 | -0.350 | 0.001 | 0.202 | 0.001 |
|  |  |  |  |  |  |  |  |  |  |  |
|  | Interaction | %*Int. | -0.064 | <0.001 | -0.048 | <0.001 | 0.335 | <0.001 | 0.195 | <0.001 |
|  |  | %*Low | -0.101 | <0.001 | -0.123 | <0.001 | 0.581 | <0.001 | 0.476 | <0.001 |
| Austria, Belgium, Switzerland, England & Wales | Education | High | 0 | ref. | 0 | ref. | 0 | ref. | 0 | ref. |
|  |  | Int. | 0.618 | <0.001 | 0.127 | 0.033 | 0.245 | 0.001 | 0.037 | 0.320 |
|  |  | Low | 0.824 | <0.001 | 0.498 | <0.001 | 0.363 | <0.001 | 0.137 | <0.001 |
|  |  |  |  |  |  |  |  |  |  |  |
|  |  | % | 0.032 | 0.468 | -0.129 | <0.001 | -0.316 | <0.001 | 0.050 | 0.143 |
|  |  |  |  |  |  |  |  |  |  |  |
|  | Interaction | %*Int. | -0.070 | <0.001 | -0.004 | 0.682 | 0.049 | 0.162 | 0.057 | 0.048 |
|  |  | %*Low | -0.062 | <0.001 | -0.032 | 0.001 | 0.110 | 0.002 | 0.180 | <0.001 |
| Turin, Slovenia, Barcelona, Basque, Madrid | Education | High | 0 | ref. | 0 | ref. | 0 | ref. | 0 | ref. |
|  |  | Int. | 0.846 | <0.001 | 0.558 | <0.001 | 0.001 | 0.983 | 0.006 | 0.809 |
|  |  | Low | 2.051 | <0.001 | 1.110 | <0.001 | -0.074 | 0.198 | 0.098 | <0.001 |
|  |  |  |  |  |  |  |  |  |  |  |
|  |  | % | 0.016 | 0.486 | -0.010 | 0.403 | -0.125 | 0.008 | -0.001 | 0.962 |
|  |  |  |  |  |  |  |  |  |  |  |
|  | Interaction | %*Int. | -0.087 | <0.001 | -0.057 | <0.001 | 0.184 | <0.001 | 0.119 | <0.001 |
|  |  | %*Low | -0.221 | <0.001 | -0.106 | <0.001 | 0.469 | <0.001 | 0.215 | <0.001 |

Negative binomial regression, controlled for age and population-period fixed effects. Coefficients for the proportion of low educated are given for a 10% difference in the proportion of low and high educated.
