# Supplementary material for: Educational expansion and inequalities in mortality—A fixed-effects analysis using longitudinal data from 18 European populations
Source: PLoS One. 2017 Aug 23;12(8):e0182526. doi: 10.1371/journal.pone.0182526 (PMC5568384; doi:10.1371/journal.pone.0182526)
Supplement: S2 Table — Adjusted for occupational class. (DOCX) [file pone.0182526.s002.docx]

**S2 Table. The association between individual education and proportion of high and low educated on all-cause mortality for men and women, 30-84 yrs. Adjusted for occupational class.**

|  |  | Men |  | Women |  |
| --- | --- | --- | --- | --- | --- |
|  |  | Coef. | p. | Coef. | p. |
| Education | High | 0 | ref. | 0 | ref. |
|  | Intermediate | 0.759 | <0.001 | 0.196 | 0.002 |
|  | Low | 0.869 | <0.001 | 0.306 | <0.001 |
|  |  |  |  |  |  |
| High education | % high | -0.033 | <0.001 | -0.015 | 0.002 |
|  |  |  |  |  |  |
|  | % high *Int. | -0.005 | 0.016 | 0.003 | 0.082 |
|  | % high *Low. | 0.002 | 0.439 | 0.012 | <0.001 |
|  |  |  |  |  |  |
| Low education | % low | -0.009 | <0.001 | -0.013 | <0.001 |
|  |  |  |  |  |  |
|  | % low *Int. | -0.008 | <0.001 | -0.002 | 0.007 |
|  | % low *Low | -0.008 | <0.001 | -0.002 | 0.006 |
